# Isolation, Potential Virulence, and Population Diversity of *Listeria monocytogenes* From Meat and Meat Products in China

**DOI:** 10.3389/fmicb.2019.00946

**Published:** 2019-05-07

**Authors:** Moutong Chen, Jianheng Cheng, Jumei Zhang, Yuetao Chen, Haiyan Zeng, Liang Xue, Tao Lei, Rui Pang, Shi Wu, Haoming Wu, Shuhong Zhang, Xianhu Wei, Youxiong Zhang, Yu Ding, Qingping Wu

**Affiliations:** ^1^Guangdong Institute of Microbiology, State Key Laboratory of Applied Microbiology Southern China, Guangdong Open Laboratory of Applied Microbiology, Guangdong Provincial Key Laboratory of Microbial Culture Collection and Application, Guangzhou, China; ^2^College of Food Science, South China Agricultural University, Guangzhou, China; ^3^Department of Food Science and Technology, Jinan University, Guangzhou, China

**Keywords:** *Listeria monocytogenes*, animal-derived food products, LIPI-3, LIPI-4, multi-locus sequence typing, antibiotic resistance

## Abstract

*Listeria monocytogenes* is a globally notorious foodborne pathogen. This study aimed to qualitatively and quantitatively detect *L. monocytogenes* from meat and meat products in China and to establish their virulence profiles and population diversity. From 1212 meat and meat product samples, 362 (29.9%) were positive for *L. monocytogenes*. Of these positive samples, 90.6% (328/362) had less than 10 MPN/g, 5.5% (20/364) samples had 10–110 MPN/g, and 3.9% (14/362) of the positive samples had over 110 MPN/g. Serogroup analysis showed that the most prevalent serogroup of *L. monocytogenes* was I.1 (1/2a-3a), which accounted for 45.0% (123/458) of the total, followed by serogroup I.2 (1/2c-3c) that comprised 26.9%, serogroup II.1 (4b-4d-4e) that comprised 4.8%, and serogroup II.2 (1/2b-3b-7) that comprised 23.3%. A total of 458 isolates were grouped into 35 sequence types (STs) that belonged to 25 clonal complexes (CCs) and one singleton (ST619) by multi-locus sequence typing. The most prevalent ST was ST9 (26.9%), followed by ST8 (17.9%), ST87 (15.3%), ST155 (9.4%), and ST121 (7.6%). Thirty-seven isolates harbored the *llsX* gene (representing LIPI-3), and they belonged to ST1/CC1, ST3/CC3, ST288/CC288, ST323/CC288, ST330/CC288, ST515/CC1, and ST619, among which ST323/CC288, ST330/CC288, and ST515/CC1 were newly reported to carry LIPI-3. Seventy-five isolates carried *ptsA*, and they belonged to ST87/CC87, ST88/CC88, and ST619, indicating that consumers may be exposed to potential hypervirulent *L. monocytogenes*. Antibiotics susceptibility tests revealed that over 90% of the isolates were susceptible to 11 antibiotics; however, 40.0% of the isolates exhibited resistance against ampicillin and 11.8% against tetracycline; further, 45.0 and 4.6% were intermediate resistant and resistant to ciprofloxacin, respectively. The rise of antibiotic resistance in *L. monocytogenes* suggests that stricter regulations should be formulated to restrict the use of antibiotic agents in human listeriosis treatment and livestock breeding.

## Introduction

*Listeria monocytogenes* is a facultative Gram-positive foodborne pathogen responsible for life-threatening listeriosis diseases, including septicaemia, meningitis, encephalitis, and miscarriage (Drevets and Bronze, 2008). The most susceptible groups are pregnant women, fetuses, elderly people, and immunocompromised individuals, who show a considerably high mortality rate (10–40%) (Hof, 2004). This pathogen can resist stressful conditions in foods and associated environments. It can grow in high salinity (10%), low temperatures (4°C), low water activity (<0.9), and a wide pH range (4.1–9.6) (Guenther et al., 2009), resulting in a wide range of habitats during different stages of the food processing. Listeriosis cases occur mainly due to the consumption of *L. monocytogenes*-contaminated foods.

Previous studies report 0.27 and 0.39 reported cases of listeriosis annually per 100,000 individuals in the United States and France, respectively (Goulet et al., 2012; Silk et al., 2012). In China, 147 clinical cases and 82 outbreak-related cases were reported from 28 provinces between 1964 and 2010 (Feng et al., 2013). In recent years, the occurrence of listeriosis diseases have been increasing in China, especially in developed cities (Wang et al., 2015, 2018). A total of 253 invasive listeriosis cases were reported between 2011 and 2016 in 19 provinces, with a fatality rate of 25.7% (Li et al., 2018b). Listeriosis has therefore become a severe public health concern to consumers in China. Although a national human listeriosis pilot surveillance was started in 2013, a risk assessment on the prevalence and characteristics of *L. monocytogenes* in foods is an urgent necessity. Although several studies have reported the presence of *L. monocytogenes* in various foods, including ready-to-eat products, mushrooms, aquatic products, meat and meat products, and frozen foods, most such studies are regionally focused (Chen et al., 2014, 2015; Liu et al., 2017; Yang et al., 2017). A comprehensive surveillance of *L. monocytogenes* in foods throughout China is of crucial importance.

Among 13 serotypes of *L. monocytogenes*, serotypes 4b, 1/2a, 1/2b, and 1/2c account for over 95% of the isolates recovered from foods and clinical cases (Orsi et al., 2011). The pathogenicity of *L. monocytogenes* may depend on the presence of virulence genes. Several virulence genes and their encoded proteins have been described in previous studies. Listeria pathogenicity island-1 (LIPI-1), along with *inlA* and *inlB*, participate in the *L. monocytogenes* infection cycle in host cells (Kreft and Vazquez-Boland, 2001). The *llsX* gene (listeriolysin S, representing LIPI-3), which encodes a haemolytic cytotoxic factor associated with the destruction of gut microbiota during infection, is mainly present in a subset of lineage I (Cotter et al., 2008; Quereda et al., 2017a,b). LIPI-3 has been detected in several sequence types (STs) of *L. monocytogenes* strains, including ST1, ST3, ST4, ST6, ST77, ST79, ST191, ST213, ST217, ST224, ST288, ST363, ST380, ST382, ST389, ST489, ST554, ST581, ST619, ST778, ST999, ST1000, and ST1001 (Chen et al., 2018b; Kim et al., 2018; Wang et al., 2018). Furthermore, a cellobiose-family phosphotransferase system with a cluster of six genes was recently identified as LIPI-4 (Maury et al., 2016). LIPI-4, which is strongly associated with neural and placental infection, was first identified as a clonal complex (CC) 4-specific virulence factor. Several STs (ST87, ST213, ST217, ST363, ST382, ST388, ST663, ST1002, ST1166, and ST619) harboring the *ptsA* gene (representing LIPI-4) have been reported in recent studies (Chen et al., 2018a; Kim et al., 2018). The pathogenic potential of *L. monocytogenes* may differ though the presence of LIPI-1, *inlA* and *inlB* gene. However, LIPI-3 (*llsX*) and LIPI-4 (*ptsA*) are strongly associated with the *L. monocytogenes* infection.

The objectives of the present study were to (i) determine the qualitative and quantitative data on *L. monocytogenes* in meat and meat products; (ii) evaluate the potential virulence and antimicrobial resistance profiles of *L. monocytogenes* isolates; and (iii) characterize the molecular serotype and genetic diversity of *L. monocytogenes* isolates recovered from the Chinese retail aquatic system. This data will be invaluable for future risk assessments.

## Materials and Methods

### Samples

Between July 2012 and April 2016, 1212 retail raw meat and meat product samples were collected from 43 cities of China, including beef (fresh = 108 samples, frozen = 46 samples), mutton (fresh = 17, frozen = 71), pork (fresh = 154, frozen = 14), minced meat (*n* = 99), preserved pork (*n* = 61), chicken (fresh = 103, frozen = 250), duck (fresh = 58, frozen = 2), dumplings (*n* = 166), steamed bun with meat (*n* = 29), wonton (*n* = 21), ham sausage (*n* = 6), and meat balls (*n* = 7). All samples were placed in insulated shipping coolers with frozen gel packs placed on the sides, middle, and top of the samples. All samples were kept below 4°C during transportation, and testing was initiated within 4 h after receiving the samples.

### Qualitative and Quantitative Analysis

Qualitative detection of *L. monocytogenes* was performed based on the National Food Safety Standard of China (4789.30-2010) (Anonymous, 2010), with minor adaptations. Briefly, 25 g of homogenized samples were added to 225 mL *Listeria* enrichment broth 1 (LB1) (Guangdong Huankai Co., Ltd., Guangzhou, China). The cultures in LB1 media were incubated at 30°C for 24 h. After incubation, 100°μL of the LB1 enrichment culture was transferred to 10 mL *Listeria* enrichment broth 2 (LB2) and incubated at 30°C for 24 h. A loopful (about 10°μL) of the LB2 enrichment culture was streaked onto *Listeria* selective agar plates (Guangdong Huankai Co., Ltd.) and incubated at 37°C for 48 h. Three to five (when possible) presumptive colonies were selected for the identification of *L. monocytogenes* using the Microgen ID *Listeria* identification system (Microgen, Camberley, United Kingdom), according to the manufacturer’s instructions.

For quantitative detection, a nine-tube most probable number (MPN) method was used, based on a previous study (Gombas et al., 2003). Briefly, nine tubes were divided into three sets of three tubes each. Homogenized samples (25 g) were added to 225 mL half frasher broth. The first set of tubes contained 10 mL of the sample homogenate in 225 mL half Frasher broth, while the second and third sets contained 10 mL of half Fraser broth (Guangdong Huankai Co., Ltd.) inoculated with 1 and 0.1 mL of the homogenate, respectively. These different volumes (10, 1, and 0.1 mL) of the sample homogenate represented 1.0, 0.1, and 0.01 g of the original sample, respectively. The nine tubes were incubated at 30 ± 2°C for 24 ± 2 h. The darkened Fraser tubes were streaked onto *Listeria* Chromagar plates. If a Fraser broth tube did not darken, it was examined again after an additional 26 ± 2 h of incubation. The presumptive pure colonies were streaked onto TSA plates and identified using the Microgen ID *Listeria* identification system. The MPN value was determined based on the number of positive tube(s) in each of the three sets and the MPN table (United States Department of Agriculture, 1998).

### Serotyping and Virulotype Determination

Multiplex PCR was used for identifying the serotypes of the 458 isolates, as described previously (Doumith et al., 2004) (Supplementary Table S1). PCR was performed in a thermal cycler (Biometra, Gottingen, Germany) with the following conditions: an initial denaturation at 94°C for 3 min; followed by 35 cycles of 94°C for 35 s, 53°C for 50 s, and 72°C for 60 s; and a final cycle of 72°C for 7 min. Two additional PCRs were performed to detect the *llsX* and *ptsA* genes (representing LIPI-3 and LIPI-4, respectively) in the *L. monocytogenes* isolates (Clayton et al., 2011; Maury et al., 2016). The PCR primers used are shown in Supplementary Table S1. The amplicons were separated on 1.5% agarose gels in TAE buffer and visualized by Goldview^®^ staining (0.005%, v/v).

### Antimicrobial Susceptibility Test

The antibiotic susceptibility of the *L. monocytogenes* isolates was determined using the KB method, according to the breakpoints for *Staphylococci* spp., as recommended by the Clinical Laboratory Standards Institute (Clinical, and Laboratory Standards Institute [CLSI], 2014) for *Staphylococcus*. The breakpoints of ampicillin and penicillin G for specific *Listeria* have been defined (M45-A2 Vol. 30 No. 18). The following 17 common antibiotic agents (disk load), including those used to treat human listeriosis, were tested: kanamycin (30°μg), gentamicin (10°μg), ciprofloxacin (5°μg), levofloxacin (5°μg), ofloxacin (5°μg), sulfamethoxazole with trimethoprim (23.75/1.25°μg), streptomycin (10°μg), rifampin (5°μg), doxycycline (30°μg), chloramphenicol (30°μg), erythromycin (15°μg), tetracycline (30°μg), meropenem (10°μg), vancomycin (30°μg), linezolid (30°μg), amoxycillin/clavulanic acid (10°μg), and sulbactam/ampicillin (10/10°μg) (Oxoid, Basingstoke, United Kingdom). Briefly, pure cultures were transferred to brain heart infusion (BHI) broth and incubated at 37°C overnight. A cell suspension was adjusted to 0.5 MacFarland standards by 0.85% NaCl (w/v). The suspension was spread onto the surface of Mueller-Hinton agar (Huankai Co., Ltd., Guangzhou). The diameters of the inhibition zones were measured using precision calipers after 24°h incubation. *Staphylococcus aureus* ATCC 25923 and *Escherichia coli* ATCC 25922 were used as quality control strains. Isolates exhibiting resistance to at least three classes of the tested antimicrobial agents were considered multidrug-resistant (Magiorakos et al., 2012).

### Multi-Locus Sequence Typing

Multi-locus sequence typing (MLST) analysis of *L. monocytogenes* was performed according to a previously published method (Ragon et al., 2008), which was based on seven house-keeping genes (*abcZ*, *bglA*, *cat*, *dapE*, *dat*, *ldh*, and *lhkA*) (Supplementary Table S2). A detailed protocol of MLST analysis, including primers, PCR conditions, STs and CCs assignments were performed according to the recommendation of Pasteur Institute website. PCR products were sequenced (Thermo Fisher Co., Ltd., Shanghai, China), and an allele number was assigned based on each variant locus of each housekeeping gene; STs and CCs were assigned *via* the *Listeria* MLST database at the Pasteur Institute website^1^. A minimum spanning tree (MST) was constructed to analyze the relationships between the isolates using the BioNumerics software Version 7.6 (Applied Maths, Belgium).

## Results

### Occurrence and Contamination Levels of *L. monocytogenes*

A total of 1212 meat and meat products (12 types) were tested in this study. As shown in Table 1, the overall prevalence of *L. monocytogenes* in meat and meat products was 29.9% (362/1212); it was detected in 45 (51.1%) mutton samples, 143 (40.5%) chicken samples, 31.3% of both minced pork (31/99) and dumpling samples (52/166), 38 (24.7%) beef samples, 6 (28.6%) wonton samples, 7 (24.1%) steamed bun with meat stuffing samples, 7 (11.7%) duck samples, 31 (18.5%) pork samples, and 1 (16.7%) ham sausage sample. Only one preserved meat sample showed the presence of *L. monocytogenes*, while no positive samples were found in meatball samples. In addition, as risk identification required quantitative data to estimate the impact of *L. monocytogenes* on consumer health, the level of contamination was also determined in the meat and meat product samples. Most samples, 90.6% (328/362) had less than 10 MPN/g, 5.5% (20/364) samples had 10-110 MPN/g, and 3.9% (14/362) of the positive samples had over 110 MPN/g, 8 of which were from chicken samples (Table 2).

**Table 1:** Positive rate of *Listeria monocytogenes* in meat and meat products.

Samples	Fresh		Frozen		Total (%)
			
	Size of sample	Positive	Size of sample	Positive	
Beef	108	17	46	21	38 (24.7)
Mutton	17	3	71	42	45 (51.1)
Pork	154	25	14	6	31 (18.5)
Minced pork	99	31	0	0	31 (31.3)
Preserved meat	61	1	0	0	1 (1.6)
Chicken	103	20	250	123	143 (40.5)
Duck	58	5	2	2	7 (11.7)
Dumplings	0	0	166	52	52 (31.3)
Steamed bun with meat stuffing	0	0	29	7	7 (24.1)
Wonton	0	0	21	6	6 (28.6)
Ham sausage	0	0	6	1	1 (16.7)
Meatball	0	0	7	0	0 (0)
Total	600	102	612	260	362 (29.9)


**Table 2:** Quantitative results of *Listeria monocytogenes* contamination in meat and meat products.

Samples	0.3 ≤ MPN < 10	10 ≤ MPN < 110	110 ≤ MPN	Total
Beef	29	9	0	38
Mutton	40	4	1	45
Pork	28	1	2	31
Minced pork	30	0	1	31
Preserved meat	1	0	0	1
Chicken	131	4	8	143
Duck	6	1	0	7
Dumpling	51	1	0	52
Steamed bun with meat stuffing	6	0	1	7
Wonton	5	0	1	6
Ham sausage	1	0	0	1
Meatball	0	0	0	0
Total	328 (90.6%)	20 (5.5%)	14 (3.9%)	362


### Serogroups

Molecular serogrouping was performed by multiplex PCR on 458 *L. monocytogenes* strains isolated from all 364 positive samples. As shown in Table 3, serogroup I.1 (1/2a-3a) was the most prevalent (45.0%). As for the other serogroups, 26.9% (123/458) of the samples were in serogroup I.2 (1/2c-3c), 4.8% (22/458) were in serogroup II.1 (4b-4d-4e), 23.3% (107/458) were in serogroup II.2 (1/2b-3b-7), and none were in serogroup III (4a-4c).

**Table 3:** Serogroup distributions of *Listeria monocytogenes* isolates.

Samples	I.1 (1/2a-3a)	I.2 (1/2c-3c)	II.1 (4b-4d-4e)	II.2 (1/2b-3b-7)	III (4a-4c)	Total
Beef	23	11	4	10	0	48
Mutton	26	22	1	8	0	57
Pork	20	9	4	7	0	40
Minced meat	20	10	2	7	0	39
Preserved meat	0	1	0	0	0	1
Chicken	86	38	8	55	0	187
Duck	4	2	0	1	0	7
Dumplings	23	23	2	15	0	63
Steamed bun with meat stuffing	3	3	0	2	0	8
Wonton	1	4	1	1	0	7
Ham sausage	0	0	0	1	0	1
Meatball	0	0	0	0	0	0
Total	206 (45.0%)	123 (26.9%)	22 (4.8%)	107 (23.3%)	0	458


### Antibiotic Susceptibility Test

All *L. monocytogenes* isolates recovered from meat and meat products were susceptible to vancomycin and amoxicillin/clavulanic acid. Over 90% of the isolates were susceptible to kanamycin, gentamicin, ofloxacin, sulfamethoxazole with trimethoprim, doxycycline, meropenem, linezolid, sulbactam/ampicillin, and penicillin. However, to some extent, *L. monocytogenes* isolates were resistant to some antibiotics, including ciprofloxacin, levofloxacin, streptomycin, rifampin, tetracycline, and ampicillin. Approximately 40.0% of the isolates exhibited resistance to ampicillin, while 8.1% were resistant to penicillin. Fifty-four (11.8%) isolates were resistant to tetracycline, and 11 exhibited intermediate resistance. In addition, 45.0 and 4.6% of the isolates were intermediate-resistant or resistant to ciprofloxacin, respectively; further, 20.3% were also intermediate-resistant to levofloxacin (Table 4). In total, 27 multidrug-resistant strains were counted.

**Table 4:** Antibiotic susceptibilities of *Listeria monocytogenes* isolates from meat and meat products.

Class of antibiotics	Antibiotics	Susceptible	Intermediate	Resistance	Susceptible (%)	Immediate (%)	Resistance (%)
Aminoglycosides	Kanamycin	≥18	14–17	≤13	432 (94.3)	4 (0.9)	22 (4.8)
	Gentamicin	≥15	13–14	≤12	439 (95.9)	8 (1.7)	11 (2.4)
Quinolones	Ciprofloxacin	≥21	16–20	≤15	231 (50.4)	206 (45.0)	21 (4.6)
	Levofloxacin	≥19	16–18	≤15	364 (79.5)	93 (20.3)	1 (0.2)
	Ofloxacin	≥16	13–15	≤12	433 (94.5)	24 (5.3)	1 (0.2)
Potentiated sulfonamide	Sulfamethoxazole with trimethoprim	≥16	11–15	≤10	438 (95.6)	4 (0.9)	16 (3.5)
Aminoglycosides	Streptomycin	≥15	12–14	≤11	183 (40.0)	246 (53.7)	29 (6.3)
Nitrofurans	Rifampin	≥20	17–19	≤16	310 (67.7)	136 (29.7)	12 (2.6)
Tetracyclines	Doxycycline	≥16	13–15	≤12	415 (90.6)	32 (7.0)	11 (2.4)
Chloramphenicols	Chloramphenicol	≥18	13–17	≤12	405 (88.4)	42 (9.2)	11 (2.4)
Macrolides	Erythromycin	≥23	14–22	≤13	403 (88.0)	39 (8.5)	16 (3.5)
Tetracyclines	Tetracycline	≥19	15–18	≤14	393 (85.8)	11 (2.4)	54 (11.8)
Novel ββ-lactam	Meropenem	≥16	14–15	≤13	453 (98.9)	0 (0)	5 (1.1)
Polypeptide	Vancomycin	≥17	15–16	≤14	458 (100.0)	0 (0)	0 (0)
Oxazolidone	Linezolid	≥21	–	≤20	455 (99.3)	0 (0)	3 (0.7)
β-lactam inhibitors	Amoxicillin/Clavulanic acid	≥20	–	≤19	458 (100.0)	0 (0)	0 (0)
β-lactam inhibitors	Sulbactam/Ampicillin	≥15	12–14	≤11	456 (99.6)	0 (0)	2 (0.4)
β-lactam	Ampicillin	<2 μg/mL	–	≥2 μg/mL	275 (60.0)	0 (0)	183 (40.0)
β-lactam	Penicillin	<2 μg/mL	–	≥2 μg/mL	421 (91.9)	0 (0)	37 (8.1)


### MLST Analysis

**FIGURE 1:**
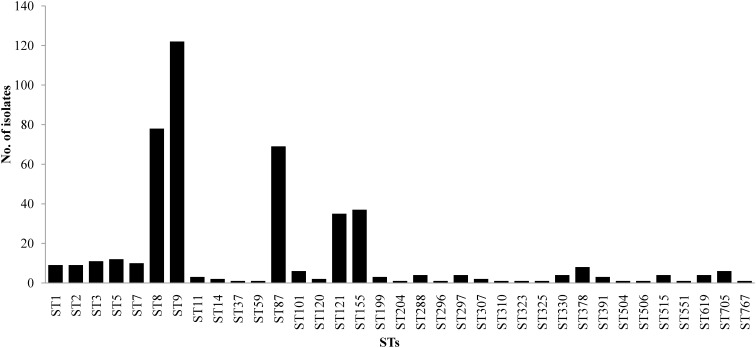
Sequence type distributions of *Listeria monocytogenes* from meat and meat products.

**FIGURE 2:**
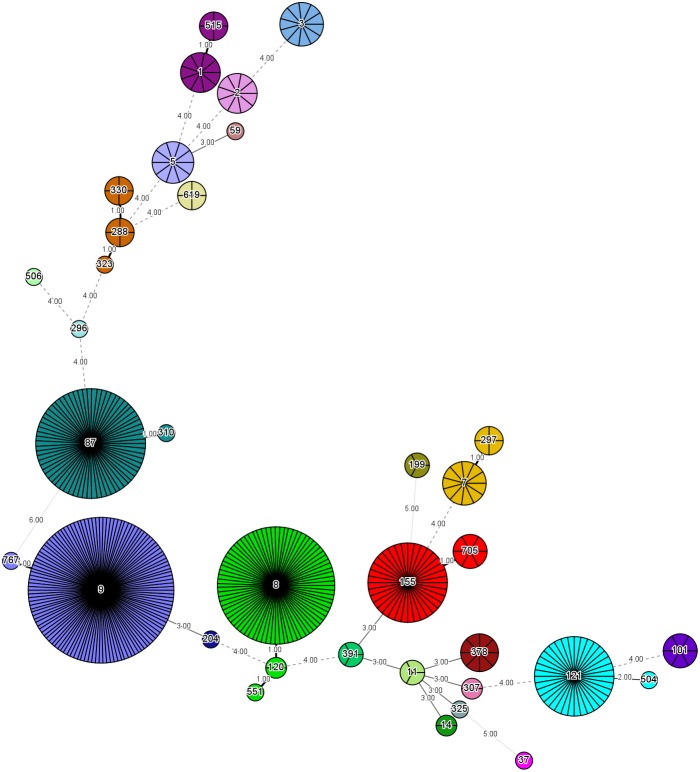
Minimum spanning tree of multi-locus sequence typing data for 458 *Listeria monocytogenes* isolates. Each circle represents one ST, and each fragment of the pie chart corresponds to a single isolate. The size of the circle is proportional to the number of isolates of that ST.

The 458 *L. monocytogenes* isolates were grouped into 35 different STs belonging to 25 CCs and one singleton (ST619) by MLST analysis (Figure 1, 2). Thirteen STs (35.1% of all STs) were represented by single isolates. Five CCs were the most prevalent: CC9 (*n* = 123 isolates; 26.9%) and CC8 (*n* = 82; 17.9%); followed by CC87 (*n* = 70; 15.3%), CC155 (*n* = 43, 9.4%), and CC121 (*n* = 35; 7.6%) (Figure 2). The remaining 20 CCs and one singleton (*n* = 105, 22.9%) were sporadically distributed (Figure 2). In addition, the presence of the *llsX* (representing LIPI-3) and *ptsA* genes (representing in LIPI-4) were also determined in the *L. monocytogenes* isolates. Thirty-seven isolates harbored *llsX*, and they belonged to ST1/CC1, ST3/CC3, ST288/CC288, ST323/CC288, ST330/CC288 ST515/CC1, and ST619; seventy-five isolates harbored *ptsA*, and they belonged to ST87/CC87, ST88/CC88, and ST619. Interestingly, isolates belonging to ST619 carried both *llsX* and *ptsA*.

## Discussion

Listeriosis is a major public health concern worldwide, with a high morbidity rate. Surveillance for *L. monocytogenes* in food items is of utmost importance for risk assessment. In this study, the contamination levels of *L. monocytogenes* in different meat and meat products in China were determined, and the phenotypic and genotypic characteristics of isolates were analyzed by serotype, antibiotic resistance, and genetic diversity. The overall prevalence of *L. monocytogenes* in meat and meat products in China was 29.9% (362/1212), similar result (26.6%) was observed conducted in Beijing city by Ma (2015) and the results from Addis Ababa, Ethiopia (Derra et al., 2013); while the different contamination rate were reported in Changchun city by Zhu et al. (2016) (43.3%) and in Liaoning province by Wen et al. (2015) (8.88%). However, other countries have been reported to have different levels of *L. monocytogenes* prevalence in meat and meat products (Ndahi et al., 2014; Ristori et al., 2014). This variation maybe attributed to differences in sample size, sample constitution, or geographical location. Among the 12 types of meat and meat products analyzed, the highest prevalence was found in mutton (51.1%), followed by chicken (40.5%) and beef (24.7%), which was consistent with the contamination reported in retail-level beef meat in Poland (Wieczorek et al., 2012). The occurrence of *L. monocytogenes* in pork (18.5%) was higher than that reported in previous studies in several countries (2.6–12.8%) (Pesavento et al., 2010; Derra et al., 2013; Li et al., 2016; Hamidiyan et al., 2018). The high prevalence of *L. monocytogenes* in meat and meat products in some countries but not others suggest meat and meat products contaminated with *L. monocytogenes* may occur at processing level, which maybe associate with the hygiene conditions of retail environments of these products. Hoelzer et al. (2012) reported that transfer probabilities of *L. monocytogenes* may be from cutting boards, scales, deli cases, deli preparation sinks to product, floor drains, walk-in cooler floors, and knife racks to food contact surfaces. In addition, it should pay attention to the persistence of *L. monocytogenes* in food production process. Simmons et al. (2014) reported that one or more PFGE types were isolated on at least three separate occasions, suggesting that the persistence of a given *L. monocytogenes* subtype in the delis. The highest prevalence of persistent predominant genotypes of *L. monocytogenes* was also observed on the Finish dairy farm with the poorest production hygiene, such as feeding surfaces, water troughs, and floors (Castro et al., 2018). Due to the high prevalence of *L. monocytogenes* in meat and meat products, some sanitization measures and regulations should be formulated to reduce the prevalence of contamination at the processing level.

Quantitative data are invaluable for estimating the impact of *L. monocytogenes* on consumer health. In the present study, the level of contamination in meat and meat products was also assessed using the MPN method. Most positive samples (90.5%) had less than 10 MPN/g, and only 3.9% of the samples had above 110 MPN/g, which were mainly chicken samples. These results were consistent with those of studies in other countries such as Poland (Modzelewska-Kapitula and Maj-Sobotka, 2014), Brazil (Ristori et al., 2014), and Ireland (Khen et al., 2015); similar results were also reported in China for other food items (Chen et al., 2018a,b), suggesting low levels of *L. monocytogenes* contamination in fresh food products. The low number of *L. monocytogenes* in most samples could still be problematic; being psychrotrophic, it may grow during the storage period. It was reported that an initial contamination by only 10 CFU/g of *L. monocytogenes* can make the food unsafe within 8 days (Salvat and Fravalo, 2004). These results demonstrate the need for further processing of meat and meat products after purchase. Additionally, cross-contamination of food items should be carefully avoided during storage to ensure food safety.

Serotyping is a classical method for *L. monocytogenes* subtyping. Our results showed that the most prevalent serotypes were serogroups I.1 (1/2a-3a), I.2 (1/2c-3c), and II.2 (1/2b-3b-7), which was consistent with the results of previous studies on *L. monocytogenes* isolated from food items (Korsak et al., 2012; Shen et al., 2013; Martin et al., 2014; Vallim et al., 2015). Interestingly, serogroup II.1 (4b-4d-4e), which we found to be scattered in all meat and meat products analyzed, has been reported to be the most predominant serogroup in ready-to-eat foods in China, including cooked meat (Chen et al., 2014; Wu et al., 2016). Serotypes 4b, 1/2b, and 1/2a have been shown to be predominant in human listeriosis cases (Orsi et al., 2011), suggesting that these isolates may exhibit pathogenicity against consumers. In fact, *L. monocytogenes* exhibit variable pathogenicity at the species-level, even though each isolate carries *inlA*, *inlB*, and virulence genes of LIPI-1.

Our MLST analysis results showed that five CCs were the most prevalent: CC9 (*n* = 123 isolates; 26.9%) and CC8 (*n* = 82; 17.9%), followed by CC87 (*n* = 70; 15.3%), CC155 (*n* = 43, 9.4%), and CC121 (*n* = 35; 7.6%). These results were consistent with those of previous studies in China (Wang et al., 2012; Li et al., 2018a), France (Felix et al., 2018), the European Union (Rychli et al., 2018), and Spain (Martin et al., 2014). The most prevalent ST was ST9 (26.9%), followed by ST8 (17.9%), ST87 (15.3%), ST155 (9.4%), and ST121 (7.6%) in this study. Thus, ST9 and ST121 may be dominant in food and food processing environments globally. In addition, ST87 has been found to be prevalent in other kinds of foods in China, including edible mushrooms and aquatic products (Chen et al., 2018a,b). ST2 and ST87 were reported to be persistent in prepacked smoked salmon in Singapore (Chau et al., 2017). To the best of our knowledge, ST87 strains are rarely reported in western countries, indicating that ST87 isolates may have a geographically associated distribution in Asia. Further surveillance should be performed for the presence of ST87 strains in food, food processing environments, and clinic cases.

*llsX* (belonging to LIPI-3) encodes a bacteriocin-like haemolytic and cytotoxic virulence factor, which plays a role in the destruction of the gut microbiota. It is critical for the establishment of infection and for the survival of the pathogen in polymorphonuclear neutrophils (Cotter et al., 2008; Quereda et al., 2016, 2017a,b). We found that 37 isolates harbored *llsX*, and they belonged to serogroups II.1 (4b-4d-4e) and II.2 (1/2b-3b-7). These isolates were present in ST1/CC1, ST3/CC3, ST288/CC288, ST323/CC288, ST330/CC288, ST515/CC1, and ST619, implying that these lineage-I isolates carrying *llsX* may be responsible for epidemic listeriosis outbreaks. ST323, ST330, and ST515 were newly reported to be *llsX*-carrying isolates. Furthermore, LIPI-4, a cluster of six genes called the cellobiose-family phosphotransferase system, was mainly involved in neural and placental infection; it was first found only in CC4 strains (Maury et al., 2016). In our results, 75 isolates carried *ptsA*, and they belonged to ST87/CC87, ST88/CC88, and ST619. Interestingly, hypervirulent CC4 strains carrying both *llsX* and *ptsA* are known to be overrepresented in human isolates (Maury et al., 2016). ST619 isolates also carried both *llsX* and *ptsA*, suggesting that they may pose a hyper-pathogenic risk to public health. In addition, one of the predominant strains found in this study, ST87, is a known epidemiological hypervirulent ST in China (Li et al., 2018b; Wang et al., 2018). Thus, potential hypervirulent isolates are present in meat and meat products.

It was reported that 162,000 tons of antibiotics was used in China in 2013, of which 84,240 tons was used for livestock breeding and cultivation (Zhang et al., 2015). The extensive use of antibiotics has facilitated the emergence of antibiotic resistance in *L. monocytogenes* (Wilson et al., 2018). The first antibiotic-resistant *L. monocytogenes* strain was isolated in 1988. An increasing number of antibiotic-resistant *L. monocytogenes* strains are being reported worldwide. Ampicillin, amoxicillin with or without gentamicin, and trimethoprim-sulfamethoxazole are the first-line therapeutic antibiotics used for listeriosis treatment (Alonso-Hernando et al., 2012). Approximately 40.0% of the strains isolated in this study were ampicillin-resistant, indicating the necessity for regular surveillances for resistance against one of the oldest antibiotics used in livestock breeding and patient treatment. In addition, 11.8% of the isolates in this study were tetracycline-resistant; this result was consistent with previous studies, in which tetracycline-resistant isolates were frequently recovered from foods (Bertrand et al., 2016; Akrami-Mohajeri et al., 2018; Noll et al., 2018). A total of 1,540 tons of tetracycline was used for humans and livestock in 2013 in China (Zhang et al., 2015); the widespread resistance may be attributed to this excessive use of tetracycline. Fluoroquinolone antibiotics are also extensively used in both human and livestock; in 2013, 25,500 tons of these antibiotics were used in China (Zhang et al., 2015). The results of this study showed that 45.0 and 4.6% of the isolates were intermediate-resistant or resistant to ciprofloxacin, respectively; further, 20.3% of the isolates were intermediate-resistant to levofloxacin (Table 4). These results were consistent with the results of previous studies (Chen et al., 2015; Wieczorek and Osek, 2017; Wilson et al., 2018). It has to be noted that intermediate-resistant strains could develop into completely resistant strains under certain circumstances (Ruiz-Bolivar et al., 2011). Several molecular mechanisms for ciprofloxacin-resistance have been documented, including gene mutations (Godreuil et al., 2003), efflux pump (Godreuil et al., 2003; Guerin et al., 2014; Bertrand et al., 2016), and plasmid-mediated resistance (Wang et al., 2003; Jacoby et al., 2006; Jiang et al., 2008). In recent years, the high prevalence of ciprofloxacin, tetracycline, and streptomycin of *L. monocytogenes* isolated from foods were reported in China (Yan et al., 2010, Yan et al., 2014), suggesting that the abuse of these antibiotics may accelerate the emergence of antibiotic resistance in *L. monocytogenes*. Although we did detect multidrug-resistant *L. monocytogenes* strains, the majority of isolates were sensitive to antibiotics commonly used in listeriosis treatment. Over 90% of the isolates were susceptible to 11 antibiotics, including kanamycin, gentamicin, ofloxacin, sulfamethoxazole with trimethoprim, doxycycline, meropenem, linezolid, sulbactam/ampicillin, penicillin, vancomycin, and amoxicillin/claulanic acid, which are commonly used to treat human listeriosis (Olaimat et al., 2018). However, the emerging threat of antibiotic resistance highlights the necessity for continuous surveillance and elucidation of molecular mechanisms behind antibiotics resistance in *L. monocytogenes* from foods, environment, and clinical cases.

In conclusion, 458 *L. monocytogenes* strains were isolated from 1212 meat and meat product samples. These strains were characterized based on serogroup, antibiotic susceptibility, and MLST. Five STs (ST8, ST9, ST87, ST155, and ST121) were predominant in meat and meat products. Several isolates carried *llsX* and/or *pstA* virulence factors, which play an important role in human listeriosis diseases, posing a potential public health concern for consumers. In addition, the rising trend of antibiotics resistance in *L. monocytogenes* suggests that strict regulations to restrict the abuse of antibiotics should be formulated urgently.

## Author Contributions

QW, JZ, and MC conceived and designed the experiments. MC, JC, and YC performed the experiments. LX, HZ, SW, RP, and HW conducted the bioinformatics analyses. MC, QW, SZ, TL, and XW drafted the manuscript. QW, YZ, and YD reviewed the manuscript. All authors read and approved the final manuscript.

## Conflict of Interest Statement

The authors declare that the research was conducted in the absence of any commercial or financial relationships that could be construed as a potential conflict of interest. The reviewer JZ declared a shared affiliation, with no collaboration, with one of the authors, YC, to the handling editor at the time of review.

## Footnotes

1 http://bigsdb.pasteur.fr/listeria/listeria.html
