# Supplementary material for: Isolation, Potential Virulence, and Population Diversity of Listeria monocytogenes From Meat and Meat Products in China
Source: Front Microbiol. 2019 May 7;10:946. doi: 10.3389/fmicb.2019.00946 (PMC6514097; doi:10.3389/fmicb.2019.00946)
Supplement: Supplementary file 1 [file Table_1.DOCX]

Table S1 Primers used for serogroups and virulence genes identification of *Listeria monocytogenes* strains

| Target gene | Forward and reverse primers (5'→3') | Specificity | Annealing temperature (°C) | Size of PCR amplicon (bp) | Reference |
| --- | --- | --- | --- | --- | --- |
| *lmo0737* | AGGGCTTCAAGGACTTACCC | *L. monocytogenes* serovars 1/2a, 3a, 1/2c and 3c | 53 | 691 | (Doumith et al., 2004) |
|  | ACGATTTCTGCTTGCCATTC |  |  |  |  |
| *lmo1118* | AGGGGTCTTAAATCCTGGAA | *L. monocytogenes* serovars 1/2c and 3c | 53 | 906 |  |
|  | CGGCTTGTTCGGCATACTTA |  |  |  |  |
| *ORF2819* | AGCAAAATGCCAAAACTCGT | *L. monocytogenes* serovars 1/2b, 3b, 4b, 4d, 4e and 7 | 53 | 471 |  |
|  | CATCACTAAAGCCTCCCATTG |  |  |  |  |
| *ORF2110* | AGTGGACAATTGATTGGTGAA | *L. monocytogenes* serovars 4b, 4d and 4e | 53 | 597 |  |
|  | CATCCATCCCTTACTTTGGAC |  |  |  |  |
| *prs* | GCTGAAGAGATTGCGAAAGAAG | All *Listeria* species | 53 | 370 |  |
|  | CAAAGAAACCTTGGATTTGCGG |  |  |  |  |
|  | TTTATCCGTACTGAAATTCC |  |  |  |  |
| *llsX* | TTATTGCATCAATTGTTCTAGGG | LIPI-3 | 52 | 200 | (Clayton et al., 2011) |
|  | CCCCTATAAACATCATGCTAGTG |  |  |  |  |
| *ptsA* | TCCTTTTTCTTTGTTGCGGA | LIPI-4 | 52 | 450 | (Maury et al., 2016) |
|  | TCTGAAGCTGTACGAAGACA |  |  |  |  |

References

Clayton, E.M., Hill, C., Cotter, P.D., and Ross, R.P. (2011). Real-time PCR assay to differentiate Listeriolysin S-positive and -negative strains of Listeria monocytogenes. *Appl Environ Microbiol* 77(1)**,** 163-171. doi: 10.1128/AEM.01673-10.

Doumith, M., Buchrieser, C., Glaser, P., Jacquet, C., and Martin, P. (2004). Differentiation of the Major Listeria monocytogenes Serovars by Multiplex PCR. *Journal of Clinical Microbiology* 42(8)**,** 3819-3822. doi: 10.1128/jcm.42.8.3819-3822.2004.

Maury, M.M., Tsai, Y.H., Charlier, C., Touchon, M., Chenal-Francisque, V., Leclercq, A., et al. (2016). Uncovering Listeria monocytogenes hypervirulence by harnessing its biodiversity. *Nat Genet* 48(3)**,** 308-313. doi: 10.1038/ng.3501.

Table S2 Primers used for multilocus sequence typing analysis

| Primer | Sequences(5’→3’) | Length (bp) | Tm (°C) |
| --- | --- | --- | --- |
| *abcZoF* | **GTTTTCCCAGTCACGACGTTGTA**TCGCTGCTGCCACTTTTATCCA | 537 | 52 |
| *abcZoR* | **TTGTGAGCGGATAACAATTT**CTCAAGGTCGCCGTTTAGAG |  |  |
| *bglAoF* | **GTTTTCCCAGTCACGACGTTGTA**GCCGACTTTTTATGGGGTGGAG | 399 | 45 |
| *bglAoR* | **TTGTGAGCGGATAACAATTT**CCGATTAAATACGGTGCGGACATA |  |  |
| *catoF* | **GTTTTCCCAGTCACGACGTTGTA**ATTGGCGCATTTTGATAGAGA | 486 | 52 |
| *catoR* | **TTGTGAGCGGATAACAATTT**CAGATTGACGATTCCTGCTTTTG |  |  |
| *dapEoF* | **GTTTTCCCAGTCACGACGTTGTA**CGACTAATGGGCATGAAGAACAAG | 462 | 52 |
| *dapEoR* | **TTGTGAGCGGATAACAATTT**CATCGAACTATGGGCATTTTTACC |  |  |
| *datoF* | **GTTTTCCCAGTCACGACGTTGTA**GAAAGAGAAGATGCCACAGTTGA | 471 | 52 |
| *datoR* | **TTGTGAGCGGATAACAATTT**CTGCGTCCATAATACACCATCTTT |  |  |
| *ldhoF* | **GTTTTCCCAGTCACGACGTTGTA**GTATGATTGACATAGATAAAGA | 453 | 52 |
| *ldhoR* | **TTGTGAGCGGATAACAATTTC**TATAAATGTCGTTCATACCAT |  |  |
| *lhkAoF* | **GTTTTCCCAGTCACGACGTTGTA**AGAATGCCAACGACGAAACC | 480 | 52 |
| *lhkAoR* | **TTGTGAGCGGATAACAATTT**CTGGGAAACATCAGCAATAAAC |  |  |
| *LhkA*-F3 | GCAAGTTTTGAATACGTATCAGCG (Lineage 3) | 480 | 52 |
| *LhkA*-R2 | TACGCATTTCATGAGAAACATCAG (Lineage 3) |  |  |
